# Temporal trends in the nutritional status of women and children under five years of age in sub-Saharan African countries: ecological study

**DOI:** 10.1590/1516-3180.2017.0267261117

**Published:** 2018-10-22

**Authors:** João Baptista Humbwavali, Camila Giugliani, Inácio Crochemore Mohnsam da Silva, Bruce Bartholow Duncan

**Affiliations:** I MSc. Nurse and Associate Professor, Instituto Superior de Ciências da Saúde, Universidade Agostinho Neto (ISCS/UAN), Luanda, Angola.; II MD, PhD. Physician and Associate Professor, Postgraduate Program on Epidemiology, Universidade Federal do Rio Grande do Sul (UFRGS), Porto Alegre (RS), Brazil.; III MSc, PhD. Physical Educator and Collaborating Professor, Center for Equity in Health, Postgraduate Program on Epidemiology, Universidade Federal de Pelotas, Pelotas (RS), Brazil.; IV MSc, PhD. Physician and Professor, Postgraduate Program on Epidemiology, Universidade Federal do Rio Grande do Sul (UFRGS), Porto Alegre (RS), Brazil.

**Keywords:** Women, Overweight, Obesity, Africa South of the Sahara

## Abstract

**BACKGROUND:**

While the global prevalence of obesity is rapidly increasing, this pandemic has received less attention in sub-Saharan Africa, particularly in the light of the persistent undernutrition that exists in the context of maternal and child health. We aimed to describe obesity trends among women of childbearing age over recent decades, along with trends in over and undernutrition among children under five years of age, in sub-Saharan African countries.

**DESIGN AND SETTING:**

Ecological study with temporal trend analysis in 13 sub-Saharan African countries.

**METHODS:**

This was a description of temporal trends in nutritional status: adult obesity, childhood overweight, low height-for-age (stunting), low weight-for-height (wasting), low weight-for-age (underweight) and low birth weight. Publicly available data from repeated cross-sectional national surveys (demographic and health surveys and multiple-indicator cluster surveys) were used. We chose 13 sub-Saharan African countries from which at least four surveys conducted since 1993 were available. We investigated women aged 15-49 years and children under five years of age.

**RESULTS:**

In multilevel linear models, the prevalence of obesity increased by an estimated 6 percentage points over 20 years among women of childbearing age, while the prevalence of overweight among children under 5 years old was stable. A major decrease in stunting and, to a lesser extent, wasting accompanied these findings.

**CONCLUSIONS:**

The upward trend in obesity among women of childbearing age in the context of highly prevalent childhood undernutrition suggests that the focus of maternal and child health in sub-Saharan Africa needs to be expanded to consider not only nutritional deficiencies but also nutritional excess.

## INTRODUCTION

Obesity, a complex condition that affects all ages and socioeconomic groups, has become one of the world’s most challenging public health problems. Because it has been raising the prevalence of noncommunicable diseases in low and middle-income countries that are still burdened with infections and nutritional deficiencies, it has helped to create a “double burden” of disease that threatens to overwhelm healthcare services.^1^^,^^2^^,^^3^ Its prevalence is increasing rapidly worldwide, and this trend is believed to be related to dietary excess, physical inactivity^4^ and increasing urbanization.^5^

Childhood overweight has also been increasing at an alarming rate. This is defined in accordance with the World Health Organization child growth standards as a weight-for-height z-score ≥ 2 standard deviations above the median of the reference population.^6^ Its worldwide prevalence increased from 4.2% (95% confidence interval, CI, 3.2%-5.2%) in 1990 to 7.8% (95% CI, 6.4%-9.1%) in 2015. This trend is expected to reach 9.1% (95% CI, 7.3%-10.9%) in 2020.^7^ Currently, at least 41 million children under five years of age are obese or overweight, and the greatest rise is being seen in low and middle-income countries.^7^ In Africa, the number of overweight children rose from 5 million in 1990 to 10 million in 2014.^8^

While tackling obesity is a current public health priority in most of the world, in sub-Saharan Africa the issue has received less attention, especially within the context of maternal and child health. In this region, the main nutritional concern continues to be undernutrition, particularly among children.

Most data available on the nutritional situation in sub-Saharan African countries are derived from standardized national surveys. However, to our knowledge, no studies focusing on sub-Saharan Africa have investigated trends using nationally representative data from several of these countries to characterize trends in excess weight among women of childbearing age and children under five years of age.

The aim of the present study was thus to describe obesity trends among women of childbearing age and overweight trends among children under five, over the past two decades, within the context of the continuing general picture of undernutrition among young children living in sub-Saharan Africa.

## METHODS

To determine temporal trends regarding the prevalence of over and undernutrition in sub-Saharan African countries, we analyzed secondary data from demographic and health surveys (DHS).^9^ These are nationally representative surveys of sizes ranging from 5,000 to 30,000 households that were funded by the United States Agency for International Development (USAID). They were usually conducted every five years to collect data on several topics from selected countries.

Standard data collection procedures and manuals had been used to guide the household survey process, and the data had been processed and presented in reports that described the situation in each country. We also used secondary data from multiple indicator cluster surveys (MICS),^10^ which are nationally representative cross-sectional household surveys that were funded by the United Nations Children’s Fund (UNICEF). These were conducted on an average of 11,000 households and provided information about maternal and child health.

For the present study, the sub-Saharan African countries from which at least four surveys providing information on the prevalence of obesity in women and/or anthropometric data on children under five years of age had been conducted since the early 1990s were eligible for inclusion. Thirteen countries were thus eligible, from which a total of 60 MICS or DHS conducted between 1993 and 2014 were available.

In the DHS, objective height and weight measurements had been made on women aged 15-49 years and on children under five years of age. Height had been measured using portable stadiometers. For children up to the age of two years, height had been measured in a lying position. Weight had been measured using portable digital scales.^11^ In MICS, similar direct measurements had been made on children, but maternal height and weight had not been obtained.^10^ Thus, the data on obesity among women of childbearing age for this study came only from the DHS.

Women were classified as obese if their body mass index was ≥ 30 kg/m^2^. In separate analyses, children under five years of age were classified with regard to wasting (low weight-for-height), stunting (low height-for-age), underweight (low weight-for-age) and overweight (high weight-for-height). These were based on z-scores for height and weight that were calculated in accordance with the World Health Organization child growth standards.^6^ Wasting, stunting and underweight were defined as two or more standard deviations below the median and overweight as two or more standard deviations above the median of the reference population.^12^ Birth weight had been obtained through interviews with parents/guardians for children under two years of age. Low birth weight was defined as < 2500 g,^13^ which is a birth weight below the third percentile, according to the World Health Organization child growth standards.

Prevalence rates, with their respective confidence intervals, were estimated for each country at each point in time, using weighting to account for aspects of the survey design, including cluster effects,^14^ in order to obtain nationally representative estimates of the population. For each indicator, temporal trends were plotted as line graphs using Microsoft Excel 2010, with one line per country. To facilitate graphical presentation of the data, the eligible countries were divided into four arbitrary groups as follows:

1. west (north): Burkina Faso, Mali, Senegal and Ivory Coast;
2. west (south): Ghana, Nigeria and Cameroon;
3. east (north): Kenya, Tanzania and Uganda; and
4. east (south): Malawi, Mozambique and Zimbabwe.

For trend analysis, estimated temporal trends were obtained for each outcome per country and for the group of 13 countries combined. Annual changes were estimated by means of linear regression using variance-weighted least squares of the values observed in each survey versus time elapsed from the first survey, using Stata, version 13.0.

Overall temporal trends regarding the prevalence of the different outcomes in the 13 countries were analyzed using a two-level hierarchical linear model. In this model, the country was the contextual variable, and both the intercept and the slope coefficient were treated as random variables. This analysis was also performed using Stata, version 13.0. Given the diversity of settings both among the countries included and among those not included in this study, we felt that treating these countries as random representatives of the sub-Saharan region was the best approach. Thus, this analysis was performed without additional weighting for country size.

All analyses were based on publicly available data from national surveys. Ethical clearance was the responsibility of the institutions that administered those surveys.

## RESULTS

**Figure 1.**
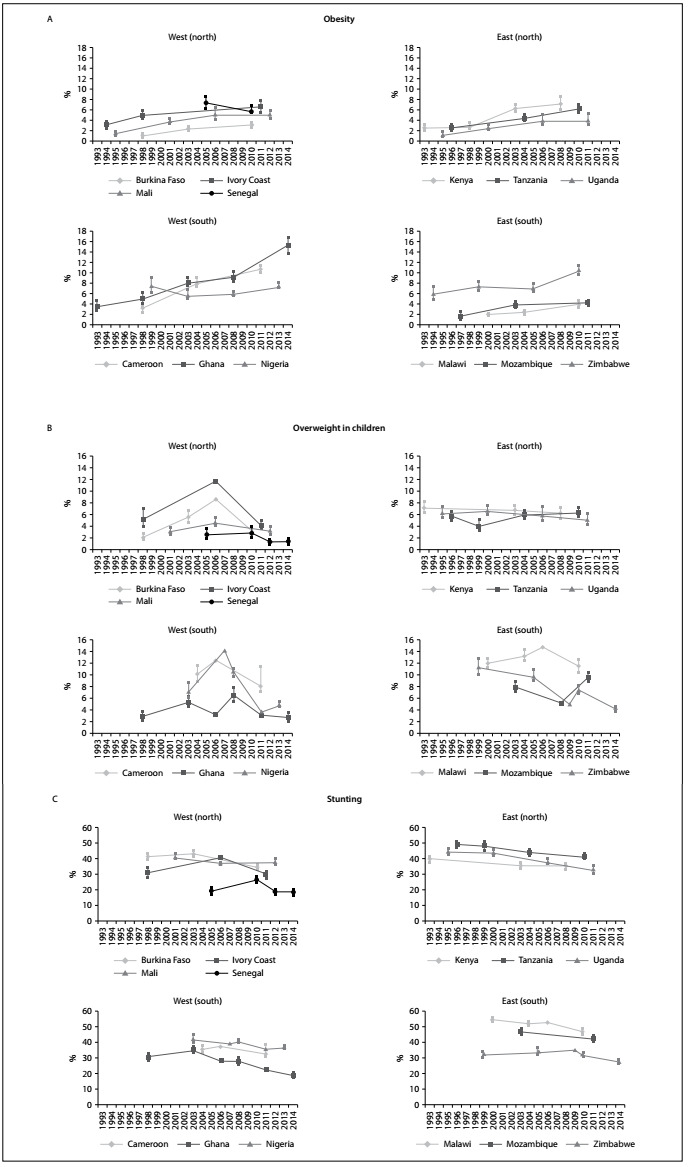
Panel A. Trends in obesity among women 15-49 years of age in 13 sub-Saharan African countries. Panel B. Trends in overweight among children under five years of age in 13 sub-Saharan African countries. Panel C. Trends in stunting among children under five years of age in 13 sub-Saharan African countries.

Sources: demographic and health surveys (DHS) (http://www.measuredhs.com/aboutsurveys/dhs/star.cfm) and multiple indicator cluster surveys (MICS) (http://www.childinfo.org/).

The trends regarding the prevalence of obesity among women aged 15-49 years are shown in Table 1 and Figure 1, Panel A. A strong upward trend in obesity can be seen among women of childbearing age in most countries, especially in Cameroon, Ghana and Kenya, with increases of 0.55, 0.48 and 0.35 percentage points per year (pp/yr), respectively (P < 0.001). In recent surveys, the prevalence of obesity among these women ranged from 3% in Burkina Faso (in 2010) to 15% in Ghana (in 2014).

**Table 1.** Trend analysis regarding obesity among women 15-49 years of age and regarding overweight and stunting among children under five years of age in 13 sub-Saharan African countries

Country	N	Prev	Last year	Change		P
				pp/yr	95% CI	
Obesity						
Burkina Faso	3	0.031	2010	0.187	0.140-0.233	< 0.001
Cameroon	3	0.107	2011	0.550	0.454-0.646	< 0.001
Ivory Coast	3	0.066	2011	0.207	0.134-0.281	< 0.001
Ghana	5	0.153	2014	0.477	0.408-0.546	< 0.001
Malawi	3	0.040	2010	0.175	0.106-0.244	< 0.001
Mali	4	0.051	2012	0.246	0.201-0.291	< 0.001
Mozambique	3	0.042	2011	0.152	0.092-0.212	< 0.001
Nigeria	4	0.075	2013	0.118	0.042-0.194	0.002
Kenya	4	0.072	2008	0.347	0.273-0.422	< 0.001
Senegal	2	0.058	2010	-0.285	-0.582-0.011	0.059
Tanzania	3	0.062	2010	0.258	0.191-0.325	< 0.001
Uganda	4	0.042	2011	0.215	0.153-0.277	< 0.001
Zimbabwe	4	0.106	2010	0.240	0.158-0.323	< 0.001
Overweight among children						
Burkina Faso	4	0.031	2010	0.088	0.024-0.152	0.007
Cameroon	3	0.082	2011	-0.441	-0.658-0.224	< 0.001
Ivory Coast	3	0.043	2011	-0.270	-0.404-0.136	< 0.001
Ghana	6	0.027	2014	-0.033	-0.091-0.026	0.275
Malawi	4	0.113	2010	0.069	-0.081-0.190	0.428
Mali	3	0.034	2012	0.020	-0.055-0.096	0.600
Mozambique	3	0.095	2011	0.132	-0.022-0.286	0.092
Nigeria	5	0.051	2013	-0.775	-0.881-0.669	< 0.001
Kenya	3	0.062	2008	-0.059	-0.145-0.027	0.176
Senegal	4	0.014	2014	-0.168	-0.270-0.067	0.001
Tanzania	4	0.064	2010	0.087	0.014-0.159	0.019
Uganda	4	0.051	2011	0.081	-0.159-0.003	0.041
Zimbabwe	5	0.050	2014	-0.507	-0.585-0.429	< 0.001
Stunting among children						
Burkina Faso	4	0.346	2010	-0.622	-0.807-0.437	< 0.001
Cameroon	3	0.325	2011	-0.573	-0.924-0.222	0.001
Ivory Coast	3	0.297	2011	-0.235	-0.520-0.051	0.107
Ghana	9	0.188	2014	-0.922	-1.070-0.774	< 0.001
Malawi	4	0.471	2010	-0.574	-0.793-0.355	< 0.001
Mali	3	0.383	2012	-0.354	-0.592-0.116	0.004
Mozambique	3	0.426	2011	-0.565	-0.848-0.282	< 0.001
Nigeria	5	0.368	2013	-0.629	-0.848-0.411	< 0.001
Kenya	3	0.353	2008	-0.329	-0.512-0.145	< 0.001
Senegal	4	0.187	2014	-0.258	-0.554-0.039	0.089
Tanzania	4	0.420	2010	-0.544	-0.725-0.363	< 0.001
Uganda	4	0.334	2011	-0.740	-0.920-0.560	< 0.001
Zimbabwe	5	0.276	2014	-0.456	-0.602-0.310	< 0.001

N = total number of surveys; pp/yr = annual percentage point change in the indicator; CI = confidence interval. Prev = prevalence at the end of the series (most recent year).

Trends regarding the prevalence of overweight among children under five years of age are also shown in Table 1 and in Figure 1, Panel B. Differences in trends between countries were evident: while some countries showed an increase in overweight among children under five, other countries showed a downward trend. Thus, there was no evident overall trend. High prevalence rates were observed in a few countries at the end of the series: 8% in Cameroon (in 2011), 11% in Malawi (in 2009) and 10% in Mozambique (in 2011).

A strong downward trend in stunting prevalence, frequently greater than 0.5 pp/yr, was observed in the sub-Saharan region (Table 1; Figure 1, Panel C). However, despite this downward trend, the prevalence rates for stunting were still high in most countries at the end of the series and were particularly notable in Nigeria, at 37% in 2013; Mali, at 38% in 2012; Tanzania, at 41% in 2010; Malawi, at 47% in 2010; and Mozambique, at 42% in 2011.

**Figure 2.**
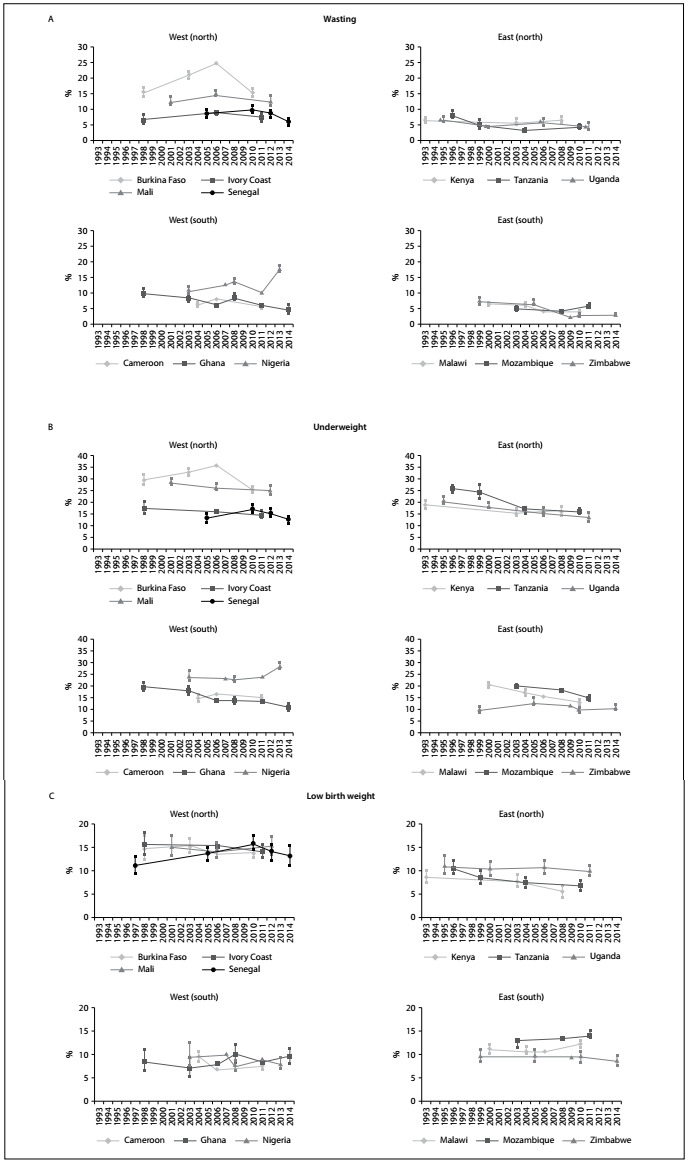
Panel A. Trends in wasting among children under five years of age in 13 sub-Saharan African countries. Panel B. Trends in underweight among children under five years of age in 13 sub-Saharan African countries. Panel C. Trends in low birth weight among children under five years of age in 13 sub-Saharan African countries.

Sources: demographic and health surveys (DHS) (http://www.measuredhs.com/aboutsurveys/dhs/star.cfm) and multiple indicator cluster surveys (MICS) (http://www.childinfo.org/).

The trends in the prevalence of wasting among children under five years of age are shown in Table 2 and Figure 2, Panel A. Most of the countries showed small downward changes, ranging from -0.01 to -0.31 pp/yr, indicating slight improvement. However, high prevalence rates were observed at the end of the series in some countries, most notably Nigeria (17%) and Burkina Faso (15%).

**Table 2.** Trend analysis regarding wasting, underweight and birth weight among children under five years of age in 13 sub-Saharan African countries

Country	N	Prev	Last year	Change		P
				pp/yr	95% CI	
Wasting among children						
Burkina Faso	4	0.154	2010	-0.060	-0.217-0.097	0.454
Cameroon	3	0.056	2011	-0.132	-0.280-0.016	0.081
Ivory Coast	3	0.076	2011	0.078	-0.068-0.225	0.295
Ghana	6	0.047	2014	-0.290	-0.377-0.203	< 0.001
Malawi	4	0.399	2010	-0.289	-0.386-0.192	< 0.001
Mali	3	0.126	2012	0.045	-0.138-0.227	0.631
Mozambique	3	0.059	2011	0.084	-0.038-0.206	0.179
Nigeria	5	0.180	2013	0.343	0.211-0.475	< 0.001
Kenya	3	0.067	2008	-0.009	-0.096-0.078	0.840
Senegal	4	0.059	2014	-0.313	-0.488-0.138	< 0.001
Tanzania	4	0.048	2010	-0.218	-0.287-0.149	< 0.001
Uganda	4	0.047	2011	-0.079	-0.158-0.001	0.047
Zimbabwe	5	0.033	2014	-0.247	-0.311-0.183	< 0.001
Underweight among children						
Burkina Faso	4	0.257	2010	-0.445	-0.631-0.260	< 0.001
Cameroon	3	0.146	2011	-0.092	-0.361-0.178	0.505
Ivory Coast	3	0.149	2011	-0.240	-0.474-0.007	0.043
Ghana	6	0.110	2014	-0.525	-0.649-0.400	< 0.001
Malawi	4	0.128	2010	-0.767	-0.930-0.604	< 0.001
Mali	3	0.255	2012	-0.347	-0.565-0.129	0.002
Mozambique	3	0.149	2011	-0.593	-0.801-0.385	< 0.001
Nigeria	5	0.287	2013	0.589	0.390-0.789	< 0.001
Kenya	3	0.161	2008	-0.212	-0.367-0.057	0.007
Senegal	4	0.126	2014	-0.152	-0.425-0.121	0.274
Tanzania	4	0.158	2010	-0.723	-0.858-0.589	< 0.001
Uganda	4	0.138	2011	-0.437	-0.572-0.301	< 0.001
Zimbabwe	5	0.112	2014	0.002	-0.088-0.093	0.958
Birth weight						
Burkina Faso	4	0.139	2010	-0.138	-0.317-0.042	0.133
Cameroon	3	0.076	2011	-0.179	-0.362-0.004	0.055
Ivory Coast	3	0.142	2011	-0.122	-0.322-0.079	0.234
Ghana	6	0.095	2014	0.096	-0.057-0.250	0.220
Malawi	4	0.123	2010	0.135	0.012-0.258	0.032
Mali	3	0.155	2012	0.031	-0.228-0.291	0.813
Mozambique	3	0.141	2011	0.126	-0.101-0.353	0.277
Nigeria	5	0.081	2013	-0.071	-0.295-0.153	0.532
Kenya	3	0.056	2008	-0.194	-0.312-0.0753	0.001
Senegal	5	0.132	2014	0.173	0.042-0.305	0.010
Tanzania	4	0.069	2010	-0.241	-0.357-0.124	< 0.001
Uganda	4	0.102	2011	-0.047	-0.170-0.076	0.452
Zimbabwe	5	0.087	2014	-0.067	-0.179-0.045	0.239

N = total number of surveys; pp/yr = annual percentage point change in the indicator; CI = confidence interval. Prev.= prevalence at the end of the series (most recent year).

Large trends with regard to underweight in children were observed in most countries in the sub-Saharan region, many decreasing more than -0.4 pp/yr (Table 2; Figure 2, Panel B). However, as with the above indicators, high rates of underweight were observed at the end of the series in some countries, with prevalence rates of more than 20% in Burkina Faso and Mali and almost 30% in Nigeria.

Regarding low birth weight (Table 2; Figure 2, Panel C), irregular annual variations characterized the trends. Some countries showed an upward trend, most notably Malawi and Senegal, with annual changes in this indicator of 0.1 and 0.2 pp/yr, respectively (P < 0.05). Prevalence rates of approximately 10% were common at the end of the series.

Table 3 summarizes the results in terms of overall tendencies based on the multilevel linear models, including all 13 countries, studied as a group. Over the period from 1993 to 2014, an average increase in obesity of 0.3 pp/yr was observed among women (or 6 percentage points over 20 years; P < 0.001), while the prevalence of overweight among the under-five children remained almost unchanged (up by 0.11 pp/yr; P = 0.14). There were significant improvements in stunting (at a mean of −0.52 pp/yr; P < 0.001) and underweight (at a mean of −0.32 pp/yr; P < 0.001). However, the other indicators of undernutrition remained basically unchanged, such that the mean change of wasting was −0.11 ­pp/­yr; P = 0.09) and the mean change in low birth weight was −0.03 pp/yr; P = 0.30).

**Table 3.** Trends in the nutritional indicators studied, in a summary analysis involving all 13 countries over the period 1993-2014

Variable	N	pp/yr	95% CI	P-value
Obesity, women 15-49 years	45	0.279	0.211-0.347	< 0.001
Overweight	51	0.101	-0.236-0.033	0.138
Stunting	51	-0.517	-0.685-0.350	< 0.001
Wasting	51	-0.107	-0.232-0.017	0.092
Underweight	51	-0.324	-0.462-0.186	< 0.001
Low birth weight	51	-0.032	-0.0908-0.028	0.296

N = total number of surveys; pp/yr = annual percentage point change in the indicator; CI = confidence interval.

A sensitivity analysis was performed within the evaluation of overall trends to investigate the effect of weighting the data so as to reflect the sizes of the different countries. The results were in the same direction and of similar magnitude, such that the increase in obesity among women was slightly lower (0.22 pp/yr), while the trend in childhood overweight was unchanged (0.11 pp/yr). The decline in stunting was slightly higher (−0.56 pp/yr), while the decrease in wasting was somewhat lower (−0.19 pp/yr). The decline in underweight was lower (−0.19 pp/yr) and not statistically significant, while the decrease in low birth weight was slightly higher (−0.09 pp/yr) and statistically significant (P = 0.02).

## DISCUSSION

In the present study, we describe a significant increase in the prevalence of obesity among sub-Saharan African women aged 15-49 years, with a rise in absolute terms of 5.6 pp over 20 years. Additionally, considering countries with at least three surveys, we found marked differences in the degree of change between countries, from 2.5 pp in Nigeria to 7.0 pp in Cameroon. The prevalence of obesity reported for these women in the most recent surveys in each country ranged from 3% to 15%.

Over the same period, the prevalence of overweight among children under five years of age lacked a clear overall trend, with an estimated average increase of only 2.0 pp over 20 years. In contrast, the prevalence of stunting decreased notably over the same period, on average by 10.3 pp. Despite this decline, the prevalence rates for stunting remained excessively high (15) (e.g. 47% and 42%, in Malawi and Mozambique, respectively). Despite the irregular trend in wasting, improvements were observed over time, as shown by the statistically significant downward trends in several countries. The prevalence of underweight also decreased significantly, down by an estimated mean of 6.5 pp over the 20-year period. Finally, favorable trends in birth weight were present in some countries, especially Kenya and Tanzania, despite the lack of improvement overall.

Within this overall picture, our findings of obesity among women are consistent with those reported by others who investigated temporal trends in overweight among women and children in low and middle-income countries using DHS. Jaacks et al. examined recent trends among women aged 19-49 years living in rural and urban areas in 33 low and middle-income countries^16^ and showed that the prevalence of overweight increased significantly in almost all countries. The increase has been most notably among urban women: 5 pp in Africa, 6 pp in the Americas and 4 pp in Asia between 1990 and 2011.^17^ The increase in prevalence reported for Africa was close to that found in the present study (5.6%), in which we considered both rural and urban areas together. Although Jones-Smith et al.^18^ observed that the prevalence of being overweight had a positive relationship with wealth and education, they noted that the rate of weight gain over time was frequently greater in groups of lower socioeconomic status. Razak et al.^19^ reported that, while populations as a whole are gaining weight, the pattern of these gains is not uniform across the range of body mass indexes. The main increases were found to be concentrated in the overweight and obese parts of the spectrum, while there was frequently little or no change in the underweight and normal-weight parts of the nutritional status distribution.

Focusing on children and adolescents (aged < 20) in developing countries, Ng et al.^20^ reported major increases in the prevalence of obesity. Thus, they suggested that once the first years of life have passed, many sub-Saharan African children manifest the result of their now obesogenic environment.

Although our findings regarding overweight in children under five years of age did not show any major trend overall, the prevalence was quite heterogeneous across nations. Some countries already had significant proportions of overweight children at the end of the period studied, whereas in others the prevalence changed little over the last two decades. Additionally, low rates of overweight in early childhood did not prevent higher rates shortly thereafter, as the previously mentioned findings of Ng et al. showed.

Although recent levels of stunting remain unacceptably high in most of the countries studied, the decline in stunting, which is a major cause of morbidity and mortality among children under five years of age,^21^^,^^22^^,^^23^ is a strong indicator that a transition in the nutritional status of under-five children is in course in sub-Saharan Africa.

The current data, within the context of the obesity pandemic and of recent trends in other developing nations,^20^ suggest that probable future improvements in indicators of childhood undernutrition in sub-Saharan Africa will be accompanied by progressive increases in childhood overweight and obesity in coming years. Moreover, the data suggest that high rates of overweight and obesity among sub-Saharan African adolescents and adults within a few decades are quite likely, even among those whose childhood was marked by wasting and stunting. Additionally, these high rates will most likely soon be accompanied by high rates of obesity-related complications, especially diabetes.

Additionally, studies have suggested that gestational diabetes, which frequently accompanies obesity during pregnancy, also increases these risks.^24^ Pre-pregnancy obesity and gestational diabetes are major risk factors for large-for-gestational-age births,^25^^,^^26^ and the risk of adult obesity is uniformly greater among those born with excess weight.^27^ This is notably so in the context of a rapid nutritional transition.^28^ Within the conceptual framework of the developmental origins of health and disease, several noncommunicable diseases originate in the fetal period and during early life.^27^^,^^29^^,^^30^ These facts highlight how important it is for sub-Saharan women to maintain a healthy weight before and during pregnancy. They also highlight the importance of avoidance of later nutritional excess among sub-Saharans who previously suffered from undernutrition during fetal life and early infancy.

In sum, these findings suggest that the time has come for public health officials in sub-Saharan African countries to implement policies for controlling obesity that include attention to women of childbearing age and young children. In global terms, overweight and obesity have already replaced undernutrition and infectious diseases as the major cause of health-related problems in the 21^st^century.^31^^,^^32^ There is little reason to believe that sub-Saharan Africa will not follow the pattern already established in most of the world’s other regions. In sub-Saharan Africa, women of childbearing age are an important target population for efforts to improve the nutritional status of future generations, since they are the key to better nutrition during fetal life and, through breastfeeding, in early childhood. For children under five years of age, growth monitoring and immunization campaigns can boost efforts to maintain balanced nutrition. Development of nutritional programs to provide better information to families about healthy eating and the importance of preventing noncommunicable diseases is also a key necessity.^30^^,^^33^ Policies to combat economic inequality and poverty, which are striking features of present-day Africa; to combat obesogenic social changes, such as increasing advertising and availability of unhealthy foods and snacks to children; to encourage physical activity; to promote family farming; to support gender equality; and to improve access to health care and education^34^ will also be beneficial in this effort. The complex challenge for many transitioning African countries will be to simultaneously address childhood undernutrition, on the one hand, and excess weight, on the other.^34^

Some limitations of our study merit comment. The group of 13 countries analyzed is not fully representative of sub-Saharan Africa, since the countries were selected based on survey availability. Therefore, caution is needed when generalizing the results. However, it is noteworthy that many of the sub-Saharan African countries with the highest obesity rates (those located in the south) were not included in our sample because they lacked a minimum number of surveys. Another limitation was our use of the body mass index to estimate cutoffs for overweight, given the widespread presence of stunting, because the progression to future overweight and obesity among children currently presenting stunting (a situation involving a large proportion of the children under five years of age in these countries) is less well understood. Nevertheless, we can highlight that one strength of our study was our use of available, high-quality data to summarize the current picture, thereby providing useful information for the public healthcare services of the countries involved.

## CONCLUSION

Our results demonstrate the presence of an epidemiologically significant upward trend in the prevalence of obesity among women of childbearing age in most of the sub-Saharan African countries included in this study. This change has been accompanied by heterogeneous, but on average relatively stable presence of overweight among children under five years of age and by significant reductions in the prevalence of chronic undernutrition. Given the context of the current obesity pandemic and the ongoing shift of disease burden to noncommunicable diseases in these countries, policymakers should place greater focus on interventions aiming to improve the nutritional status of women of childbearing age and children during the first years of life in sub-Saharan Africa, not only to eradicate states of nutritional deficiency but also to prevent states of nutritional excess.
